# Association of Superficial and Deep Macular Microvasculature with Central Visual Field Sensitivity in Glaucomatous Eyes with High Myopia

**DOI:** 10.3390/jcm11154430

**Published:** 2022-07-29

**Authors:** Anna Lee, Joong Won Shin, Jin Yeong Lee, Min Su Baek, Michael S. Kook

**Affiliations:** Department of Ophthalmology, College of Medicine, University of Ulsan, Asan Medical Center, Seoul 05505, Korea; hooni811@naver.com (A.L.); sideral@hanmail.net (J.W.S.); gnyoungee@naver.com (J.Y.L.); bmasiuenk@daum.net (M.S.B.)

**Keywords:** glaucoma, high myopia, macular vessel density, superficial vascular plexus, deep vascular plexus

## Abstract

Purpose: To investigate the relationship between two distinct layers of macular vessel density (superficial vascular plexus (SVP) and deep vascular plexus (DVP)) and central visual field sensitivity (cVFS) in open-angle glaucoma (OAG) eyes with high myopia. Method: This retrospective cross-sectional study included 148 OAG eyes (64 highly myopic (HMG) and 84 non-highly myopic glaucomas (NMG)) as well as 54 healthy eyes. High myopia was defined as a spherical equivalent of less than −6.0 diopters or an axial length of ≥26.0 mm. The global and sectoral SVP–cVFS and DVP–cVFS relationships were compared in each group. Macular ganglion cell-inner plexiform layer thickness (mGCIPLT)–cVFS relationships were also investigated as reference standards. Linear regression analysis was performed to identify the clinical factors associated with cVFS. Results: DVP–cVFS correlations were as strong as those for SVP–cVFS and mGCIPLT–cVFS in HMG eyes. In contrast, DVP–cVFS correlations were significantly lower than SVP–cVFS and mGCILT–cVFS correlations in NMG eyes. In linear regression analysis, both SVP and DVP were significantly associated with cVFS in HMG eyes, but only SVP showed a significant correlation with cVFS in NMG eyes. Conclusion: DVP assessment using OCT-A may be a useful tool for detecting and monitoring OAG eyes with high myopia.

## 1. Introduction

Evaluations of structure–function relationships in glaucoma are crucial for estimating the severity of this disease and for differentiating true glaucomatous damage from testing artifacts [1,2]. Although there is a moderate relationship between structure and function in glaucoma [3,4], the association between the retinal nerve fiber layer (RNFL) thickness and visual field (VF) sensitivity is relatively weak in glaucomatous eyes with high myopia, as morphological alterations in the optic nerve head (ONH) and/or RNFL, including parapapillary atrophy, tilted disc configuration, and/or stretching/distortion of the RNFL, may render the structural evaluation imprecise in highly myopic eyes [5,6,7]. In this regard, there is a clinical need for an alternative biomarker to RNFL thickness (RNFLT) in detecting and monitoring glaucomatous damage in highly myopic eyes.

Optical coherence tomography angiography (OCT-A) is a new methodology that has enabled a non-invasive assessment of the microvasculature in the ONH, peripapillary retina, and macula. Currently, OCT-A provides two-layer segmentation slabs in the macula, consisting of the superficial (SVP) and deep vascular plexus (DVP) based on the anatomical array. Recent studies have indicated that SVP vessel densities (VDs) have better diagnostic accuracy for detecting glaucomatous damage than DVP VDs [8,9]. However, a DVP VD reduction is frequently found in eyes with high myopia and occurs significantly more quickly in open-angle glaucoma (OAG) eyes with high myopia than in those without high myopia [10,11]. These findings suggested that the DVP VD can be a potential biomarker for detecting and monitoring glaucomatous damage in highly myopic eyes. Nonetheless, little is known about the relationship between macular VDs (mVDs) derived from DVP and VF sensitivity in highly myopic OAG eyes. The aim of our present study was to investigate the global and regional correlations between mVDs derived from the SVP and DVP using OCT-A and central VF sensitivity (cVFS) data assessed by standard automated perimetry (SAP) in OAG eyes with and without high myopia. In addition, the correlation between macular ganglion cell-inner plexiform layer thickness (mGCIPLT) and cVFS was also assessed in each OAG group as the reference standard.

## 2. Methods

### 2.1. Study Subjects

This retrospective cross-sectional study was approved by the Institutional Review Board (IRB) of Asan Medical Center and all procedures conformed to the tenets of the Declaration of Helsinki. The requirement for informed consent from the study subjects was waived by the IRB due to the retrospective study design (Approval code: 2018-1008).

The medical records of patients who visited our glaucoma clinic at Asan Medical Center from November 2016 to April 2021 were consecutively reviewed. All patients underwent an initial comprehensive ophthalmic work-up including a review of their medical history, a measurement of best-corrected visual acuity (BCVA), an intraocular pressure (IOP) measurement using Goldmann applanation tonometry, slit-lamp biomicroscopy, refractive error analyses with an autorefractor (KR-890; Topcon, Tokyo, Japan), axial length (AL) measurements (IOL Master version 5; Carl Zeiss Meditec, Dublin, CA, USA), ultrasound pachymetry (Tomey SP-3000, Nagoya, Japan) for central corneal thickness (CCT) measurement, dilated color fundus photography (Canon, Tokyo, Japan), optic disc stereoscopic photography, red-free RNFL photography (Canon), Humphrey field analyzer (HFA) Swedish Interactive Threshold Algorithm (SITA)-Standard 24-2 VF testing (Carl Zeiss Meditec), spectra-domain optical coherence tomography (SD-OCT; Cirrus HD; Carl Zeiss Meditec), and imaging with a commercial OCT-A system (Angiovue; Optovue, Inc., Fremont, CA, USA).

Our current study enrolled OAG cases as well as normal healthy subjects, both of which had to meet the following inclusion criteria: age ≥ 18 years, BCVA ≥ 20/30, reliable VF testing (false-positive errors < 15%, false-negative errors < 15%, and fixation loss < 20%), normal anterior and posterior chamber, and open angles on slit lamp exam and gonioscopy. In addition, OAG subjects were required to have the presence of a glaucomatous optic disc change (i.e., focal or generalized neural rim loss and localized or diffuse atrophy of the RNFL) with compatible glaucomatous VF defects [12] irrespective of the IOP level. To account for the learning effects of VF testing, a second VF test was obtained within one week if the first VF result was judged to be glaucomatous. Subsequently, enrolled OAG patients were classified into two groups: highly myopic [HMG] vs. non-highly myopic glaucoma [NMG], in which high myopia was defined as a spherical equivalent (SE) less than − 6.0 diopters with an AL ≥ 26 mm [13,14]. The normal healthy participants consisted of subjects from the general eye clinic that were required to have (1) an IOP < 21 mmHg with no history of an elevated IOP; (2) a normal-appearing ONH with normal SD-OCT results based on the normative database (no RNFL defects); and (3) normal VF test results (i.e., a PSD within 95% confidence limits and a GHT result within normal limits) [12].

Subjects were excluded if they had one or more of the following conditions: severe posterior pole changes and/or posterior staphyloma that may impair adequate SD-OCT/OCT-A evaluations, a history of intraocular surgery (including any cataract/glaucoma operations), a history of ocular trauma, a history of systemic or neurologic conditions, or any other ophthalmic diseases that could affect ONH/VF evaluations, including retinal detachment, diabetic retinopathy, or retinal vascular occlusive diseases. Patients who were receiving topical anti-glaucoma eye drops or systemic hypotensive medications were not excluded, however. If both eyes were eligible in any subject, one eye was randomly selected for the analysis.

### 2.2. SD-OCT Imaging

The macular ganglion cell-inner plexiform layer thickness (mGCIPLT) was measured at the macular region, centered on the fovea, using Cirrus HD SD-OCT software, version 10.0 (Carl Zeiss Meditec, Jena, Germany). The mGCIPLT was measured within an annulus with inner vertical and horizontal diameters of 1 and 1.2 mm, and outer vertical and horizontal diameters of 4 and 4.8 mm, respectively. The mGCIPLT was also measured globally and at six sectors (superotemporal (ST), superocenter (SC), superonasal (SN), inferonasal (IN), inferocenter (IC), inferotemporal (IT)). All SD-OCT images were reviewed by three glaucoma specialists (A.L., J.Y.L. and M.S.K.) for an evaluation of their quality. Only images with a signal strength >7 were included in the analysis. SD-OCT images were excluded if they had (1) motion artifacts, (2) poor centering on the fovea, or (3) segmentation errors.

### 2.3. OCT-A Imaging

OCT-A imaging of the macular regions was performed using the AngioVue OCT-A system (Optovue Inc). This OCT-A system utilizes a motion contrast technique optimized for the split-spectrum amplitude-decorrelation angiography method to demonstrate the macular microvasculature in different layers [15]. All OCT-A parameters from this present study were extracted from the same version of AngioVue software (version 2018.1.0.43) to ensure data consistency. mVD measurements were calculated from a 6 × 6 mm^2^ region centered on the fovea. All macular OCT-A en-face images were automatically segmented into the slabs of SVP (from the internal limiting membrane to the posterior boundary of the inner plexiform layer) and DVP (from the posterior margin of the inner plexiform layer to posterior boundary of the outer plexiform layer). In our present analyses, the projection artifact resolved (PAR) software was used to obtain DVP images in which the projection artifacts from the retinal vessels of overlying superficial layer were automatically removed. All OCT-A images were reviewed by three glaucoma specialists (A.L., J.Y.L. and M.S.K.) for an evaluation of image quality. OCT-A images were excluded if they had (1) poor image quality with signal strength index <48; (2) motion artifacts (i.e., significant residual motion line); (3) localized weak signal intensity caused by vitreous floaters; poor clarity (i.e., media opacity); (4) images not centered on the fovea (i.e., fixation error); or (5) segmentation failure. Eligible images were then imported into a computer program written using MATLAB software (The MathWorks, Natick, MA, USA) and mVD values (SVP and DVP) were calculated using the method described by Shin et al. [16,17]. Briefly, the en-face SVP and DVP layer images were binarized using an automated thresholding algorithm. The vascularization area was denoted by the white pixels and the VD was defined as the percentage of white pixels relative to the total number of pixels within the region of interest. AL-related magnification effects were not considered in the present study since the parafoveal area has a relatively uniform vascular network and the VD measurement in this area is minimally affected by ocular magnification related to axial elongation [18]. SVP and DVP mVDs were calculated in the same sectors as mGCIPLT measurements within an annulus of 1 and 1.2 mm inner vertical and horizontal diameters, and 4 and 4.8 mm outer vertical and horizontal diameters, respectively.

### 2.4. Structure–Function and Vasculature–Function Correlations

**Figure 1:**
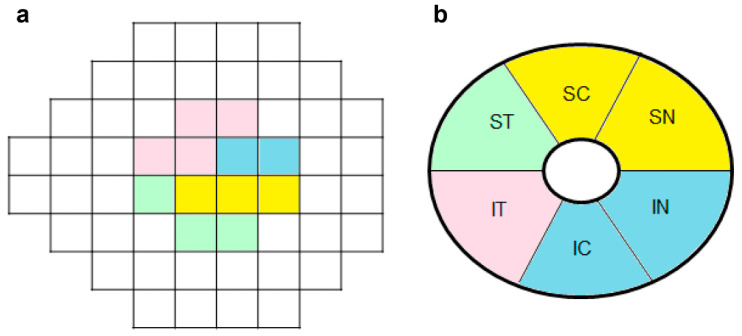
Structure– and vasculature–function correspondence map: (**a**) Central Visual field mean sensitivities (cVFS) in the 12 central points of the 24-2 VF test were grouped into four sectors and correlated with (**b**) corresponding regional macular ganglion cell-inner plexiform layer thickness and macular vessel density according to Garway-Heath et al. [21]. cVFS central visual field mean sensitivity, IC inferocenter, IN inferonasal, IT inferotemporal, SC superocenter, ST superotemporal, SN superonasal.

Structure– and vasculature–function correlations were analyzed by comparing the mGCIPT and mVD, and the corresponding cVFS values measured by SAP. The cVFS in the 12 central points of the SITA-Standard 24-2 test, which topographically corresponds to the macular region within 4.8 mm of the center of the fovea, was calculated by switching from a logarithmic dB to a nonlogarithmic dB (10 × log (1/Lambert); 1/L scale) and averaging the VF points in a given sector [19,20]. The cVFS was grouped into four sectors topographically, according to the modified method described by Garway-Heath et al. [21]. As shown in Figure 1a,b, the ST cVFS was defined as the average sensitivity of two ST VF points (paired with IN + IC mGCIPLT or IN + IC mVD) and the SN cVFS was defined as the average sensitivity of four SN VF points (paired with IT mGCIPL or IT mVD). The IN cVFS was defined as the average sensitivity of three IN VF points (paired with ST mGCIPLT or ST mVD) and the IT cVFS was defined as the average sensitivity of three IT VF points (paired with SN + SC mGCIPLT and SN + SC mVD) [17,22].

### 2.5. Statistical Analysis

All statistical analyses were performed using SPSS version 21.0 (IBM Corp., Chicago, IL, USA) and *p*-values less than 0.05 were considered statistically significant. Results are presented as either mean values with a standard deviation (SD) or as a frequency and percentage. The normality of distribution was assessed using the Kolmogorov–Smirnov test. The demographic and clinical characteristics of the study subjects were compared among the HMG, NMG, and healthy control groups. Comparisons among groups were performed using one-way analysis of variance with Tukey’s post-hoc test for quantitative variables and Chi-square test with the Bonferroni correction for categorical variables. The correlations of the average/sectoral mGCIPLT and SVP and DVP mVD values with the cVFS were evaluated using linear regression analysis [23,24]. In the regression analyses, the cVFS was regarded as the dependent variable and the average/sectoral mGCIPLT, SVP and DVP mVD values were independent variables. The Steiger test was performed to determine whether there was a significant difference in the correlation coefficients among mGCIPLT-cVFS, SVP mVD-cVFS, and DVP mVD-cVFS relationships [25]. In the multiple comparison, we applied Bonferroni corrections to obtain adjusted *p*-values. Thus, significance level was set at 0.017 (adjusted *p*-value = 0.05 divided by 3) in each comparison among correlation coefficients. Clinical variables associated with the cVFS were also analyzed using univariable and multivariable linear regression analysis in each OAG subgroup as well as in the entire OAG group. Variables with *p* values < 0.05 in the univariable analysis were included in the multivariable linear regression analyses with a stepwise elimination process. To avoid the possible multi-collinearity effects between the SVP mVD and DVP mVD, two different multivariable models were separately constructed.

## 3. Results

After an initial review of our hospital database, a total of 224 eyes consisting of 59 healthy eyes and 165 OAG eyes (73 HMG and 92 NMG) were identified and initially enrolled in our study cohort. Of these enrolled cases, three eyes with unreliable VF testing and 19 eyes with poor quality SD-OCT/OCT-A scans were excluded despite image reacquisition on the same day. Hence, our final cohort comprised 202 eyes consisting of 54 healthy eyes, 64 HMG eyes, and 84 NMG eyes. HMG eyes showed a significantly longer AL and lower SE compared to the NMG and healthy eyes (Table 1, *p* < 0.05). Compared to the healthy control eyes, HMG eyes showed significantly lower global and regional mGCPILT, SVP and DVP mVD values (all *p* < 0.05), while NMG eyes showed significantly lower global and regional mGCIPLT and SVP mVD values (all *p* < 0.05). However, there was no significant differences found in the global and regional DVP mVD values between NMG and healthy eyes (all *p* > 0.017). In the comparisons between HMG and NMG eyes, the HMG eyes showed significantly lower global and regional mGCIPLT, SVP and DVP mVDs than the NMG eyes (*p* < 0.05, except for IT mGCIPLT). There were no significant differences in age, CCT, or frequency of hypertension or diabetic mellitus among the three groups (*p* > 0.05).

Pairwise comparisons between structure–function and vasculature–function correlations in the HMG and NMG eyes are presented in Table 2. The entire group showed statistically significant structure– and vasculature–function correlations in both the global and regional assessments (all *p* < 0.05). Globally, no significant differences between the mGCIPLT–cVFS and SVP mVD–cVFS correlations were found (r = 0.483 vs. 0.509, *p* = 0.669), while the DVP mVD–cVFS correlation was weaker than that of the mGCIPLT–cVFS with a borderline *p*-value (r = 0.483 vs. 0.307, *p* = 0.039) or SVP–cVFS (r = 509 vs. 0.307, *p* < 0.001). In terms of regional relationships, the correlation coefficients for SVP mVD–cVFS were similar with those for mGCIPLT–cVFS (ST: r = 0.502 vs. 0.350, *p* = 0.035; SC+SN: r = 0.462 vs. 0.476, *p* = 0.840; IC+IN: r = 0.466 vs. 0.441, *p* = 0.708; IT: r = 0.622 vs. 0.563, *p* = 0.297), whereas DVP mVD–cVFS correlations were significantly weaker than the mGCIPLT–cVFS correlations in all four sectors (ST: r = 0.502 vs. 0.253, *p* = 0.005; SC+SN: r = 0.462 vs. 0.241, *p* = 0.013; IC+IN: r = 0.466 vs. 0.219, *p* = 0.005; IT: r = 0.622 vs. 0.301, *p* < 0.001), and the SVP mVD–cVFS correlations except for the ST sector (*p* < 0.05). (ST: r = 0.350 vs. 0.253, *p* = 0.050; SC+SN: r = 0.476 vs. 0.241, *p* = 0.001; IC+IN: r = 0.441 vs. 0.219, *p* < 0.001; IT: r = 0.563 vs. 0.301, *p* < 0.001).

In the subgroup analysis, HMG eyes showed statistically significant structure– and vasculature–function correlations following global and regional assessments (*p* < 0.001). Notably, there were no significant differences observed in the global and regional correlation coefficients between the DVP mVD–cVFS and mGCIPLT–cVFS (Global: r = 0.537 vs. 0.508 *p* = 0.795; ST: r = 0.507 vs. 0.467, *p* = 0.734; SC+SN: r = 0.568 vs. 0.423, *p* = 0.207; IC+IN: r = 0.441 vs. 0.403, *p* = 0.950; IT: r = 0.535 vs. 0.454, *p* = 0.520), or between the DVP mVD–cVFS and SVP mVD–cVFS (Global: r = 0.572 vs. 0.508, *p* = 0.284; ST: r = 0.535 vs. 0.467, *p* = 0.220; SC+SN: r = 0.501 vs. 0.423, *p* = 0.256; IC+IN: r = 0.512 vs. 0.403, *p* = 0.121; IT: r = 0.518 vs. 0.454, *p* = 0.309).

NMG eyes showed statistically significant structure– and vasculature–function relationships in the global assessment (*p* < 0.05). While there was no significant difference found between the mGCIPLT–cVFS and SVP mVD–cVFS correlation (r = 0.552 vs. 0.535, *p* = 0.835), the DVP mVD–cVFS correlation was weaker than that of the mGCIPLT–cVFS with borderline p-values (r = 0.552 vs. 0.270, *p* = 0.030) and SVP mVD–cVFS in NMG eyes (r = 0.535 vs. 0.270, *p* = 0.001) globally. Regionally, the structure– and vasculature–function relationships were all statistically significant (*p* < 0.05), except for those at the ST sector (r = 0.176, *p* = 0.109 for ST SVP mVD; and r = 0.076, *p* = 0.493 for ST DVP mVD) and SC+SN sector (r = 0.164, *p* = 0.137 for SC+SN DVP mVD). In the comparisons of structure– and vasculature–function relationship, the correlation coefficients for the DVP mVD–cVFS were significantly lower than those for the mGCIPLT–cVFS (ST: r = 0.508 vs. 0.076, *p* = 0.002; SC+SN: r = 0.499 vs. 0.164, *p* = 0.015; IC+IN: r = 0.631 vs. 0.259, *p* = 0.002; IT: r = 0.728 vs. 0.327, *p* < 0.001) and SVP mVD-cVFS, except for the ST sector. (ST: r = 0.176 vs. 0.076, *p* = 0.272; SC+SN: r = 0.498 vs. 0.164, *p* < 0.001; IC+IN: r = 0.490 vs. 0.259, *p* = 0.010; IT: r = 0.702 vs. 0.327, *p* < 0.001)

Table 3 presents the results of univariable and multivariable linear regression analysis to determine the clinical factors associated with cVFS in the entire HMG and NMG groups. Multivariable analyses were separately performed with two models to avoid multi-collinearity between the SVP mVD and DVP mVD measurements. (i.e., entire group: SVP mVD vs. DVP mVD [r = 0.764, *p* < 0.001], HMG group: SVP mVD vs. DVP mVD [r = 0.840, *p* < 0.001], NMG group: SVP mVD vs. DVP mVD [r = 0.653, *p* < 0.001]) Multivariable linear regression analysis of the entire study cohort (*n* = 148) revealed that age, mGCIPLT, and SVP mVD were significantly associated with the cVFS (*p* < 0.05). In subgroup analysis, age, mGCIPLT, and SVP mVD were the factors significantly associated with cVFS in NMG eyes (*p* < 0.05), whereas age, mGCIPLT, SVP mVD, and DVP mVD were significantly associated with the cVFS in HMG eyes (*p* < 0.05).

**Figure 2:**
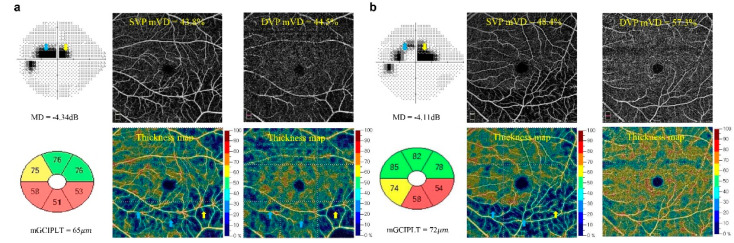
Two representative cases with early-stage open-angle glaucoma and a different axial length (AL): (**a**) A high myopia case with an AL of 26.44 mm. (**b**) A non-high myopia case with an AL of 24.80 mm. The visual field mean deviation was −4.34 decibel (dB) ((**a**), top left) and −4.11 dB ((**b**), top left), respectively. The average macular ganglion cell-inner plexiform layer thickness (mGCIPLT), assessed by spectral-domain optical coherence tomography, was 65 μm ((**a**), bottom left) and 72 μm ((**b**), bottom left), respectively. The superficial vascular plexus (SVP) macular vessel density (mVD) loss, as determined by the optical coherence tomography angiography, showed a good topographic correlation with the central visual field loss in both cases ((**a**), top middle and bottom middle; (**b**), top middle and bottom middle) based on the en-face image and thickness map. A deep vascular plexus (DVP) mVD loss was detected in the inferior macula, which corresponded to the central visual field loss in high myopia case ((**a**), top right and bottom right). By contrast, there was no definitive DVP mVD loss in the non-high myopia case ((**b**), top right and bottom right). Colored arrows indicate visual field loss corresponding to SVP and DVP mVD loss. AL axial length, dB decibel, DVP deep vascular plexus, mGCIPLT macular ganglion cell-inner plexiform layer thickness, mVD macular vessel density, SVP superficial vascular plexus.

Two representative HMG and NMG cases (a, b) are presented in Figure 2 to demonstrate a high correlation of DVP mVD–cVFS relationships in the HMG eyes, as opposed to a poor correlation of DVP mVD–cVFS relationship in the NMG eyes. Figure 2a shows a representative HMG case with an AL of 26.44 mm and a VF MD of −4.34 dB. Figure 2b presents a representative NMG case with an AL of 24.80 mm and a VF MD of −4.11 dB. The average mGCIPLT, SVP mVD, and DVP mVD values in the HMG eye were 65 µm, 43.8% and 44.5%, and those of the NMG eye were 72 µm, 48.4% and 57.3%, respectively. The SVP mVD loss was topographically well correlated with VF loss according to the en-face image and thickness map in both the HMG and NMG cases. The blue arrow in the figures indicates the ST sector within the central 10° VF area, corresponding to the IC+IN sector of the SVP mVD in the thickness map, and the yellow arrow indicates the SN sector within the central 10° VF area corresponding to the IT sector of the SVP mVD in the thickness map. In contrast, while DVP mVD loss was well detected in the inferior macular region of the thickness map, which corresponds to the central VF defect in the representative HMG case (Figure 2a), there was no definitive DVP mVD loss according to the en-face image and thickness map in the NMG case (Figure 2b).

## 4. Discussion

High myopia is a well-known risk factor for glaucoma and its severity shows a positive correlation with the probability of developing this disorder [26,27,28]. The findings of recent studies suggest that peripapillary VD measurements using OCT-A may be less affected by myopic structural alterations of the ONH/RNFL, which contributes to a stronger correlation with VF sensitivities than peripapillary RNFLT in highly myopic glaucomatous eyes [29,30]. Hence, the assessment of vasculature–function relationships in glaucomatous eyes with high myopia is of great clinical relevance. Of note, in particular, knowledge of the relationship between the mVD and cVFS is critical in glaucoma as the macular structure is vital for vision-related daily activities [31]. In our present study, while the DVP mVD–cVFS correlation was significantly weaker than that of either the SVP mVD–cVFS or mGCIPLT–cVFS in the NMG eyes, there were no significant differences in the global and regional correlation coefficients between the DVP mVD–cVFS and mGCIPLT–cVFS, or between the DVP mVD–cVFS and SVP mVD–cVFS in the HMG eyes. Multivariable regression analyses revealed that DVP mVD as well as SVP mVD was significantly associated with the cVFS in HMG eyes, while only SVP mVD was significantly related to the cVFS in NMG eyes. To our knowledge, this present study is the first to evaluate the relationship between the cVFS and mVDs of two distinctive layers in glaucomatous eyes with high myopia.

Previous studies have indicated that the SVP mVD loss is greater than the DVP mVD loss in eyes with early-stage glaucoma [8,15]. Rao et al. [15] reported that the SVP mVD had significantly decreased in glaucomatous eyes compared with healthy controls. Moreover, Lee et al. [8] demonstrated a lower SVP mVD in early-stage glaucomatous eyes (MD ≥ −6 dB) compared to healthy eyes, but no significant difference in the DVP mVD between these two groups. Our current findings are consistent with those of previous studies [8,15] in that our NMG eyes (MD = −5.59 dB) showed a significantly lower SVP mVD compared to healthy controls (*p* < 0.05), while there was no significant difference in the DVP mVD between the two groups. In contrast, the HMG eyes in our study showed significant DVP mVD as well as SVP mVD reductions, resulting in significantly lower global and regional DVP mVD values compared to the healthy eyes (Table 1, *p* < 0.05). An explanation of our present results can possibly be found in the recent report by Ye et al. [10], showing that a decreased DVP mVD was significantly associated with the outer retinal layer alteration in high myopia. Cheung et al. [32] also demonstrated that a deep perifoveal VD was mostly associated with AL and SE in a group of 145 healthy eyes with SE ranging from +0.50 to −16.50 diopters. Our current data indicate that while a DVP mVD reduction may be rare in NMG eyes, it may be frequently observed in the HMG eyes, indicating that this reduction is closely related to axial elongation in HMG eyes. The global and regional DVP mVD-cVFS correlations we observed were comparable to the SVP mVD–cVFS or mGCIPLT–cVFS correlations in HMG eyes, whereas they were significantly weaker than the SVP mVD–cVFS and mGCIPLT–cVFS correlations in NMG eyes (Table 2). Moreover, while both the HMG and NMG groups showed similar SVP mVD–cVFS correlations (global r = 0.572 vs. 0.535 for HMG vs. NMG), the HMG eyes in our study series showed generally higher global and regional DVP mVD–cVFS correlations (r = 0.508; global) compared to NMG eyes (r = 0.270; global; Table 2). Our findings suggest that a deep layer mVD reduction In addition to superficial mVD and/or mGCIPLT loss may be considered to detect and monitor glaucomatous damage in eyes with high myopia.

There are several possible explanations for our findings that HMG eyes showed higher global and regional DVP mVD–cVFS correlations compared to NMG eyes. First, although recently updated OCT-A software provides PAR algorithms that minimize the projection flow from the superficial retinal layer, projection artifacts may not be completely removed during the DVP mVD measurements. Since the denser SVP mVD of NMG eyes may induce greater flow projection to the deep layer compared to that of HMG eyes, the DVP mVD measurements in HMG eyes may have been less affected by these projection artifacts than those in NMG eyes. Less amount of projection artifacts in the DVP may result in greater mVD-cVFS correlations in the HMG eyes than in the NMG eyes. Second, the DVP is more vulnerable than the SVP to the blood flow impairment associated with axial elongation due to their different anatomical locations. The DVP is located more remotely and distally from larger retinal vessels [33] and is thereby subject to poor recovery from a VD loss, which may make it more vulnerable to blood flow reduction. Third, retinal tissue thinning associated with ocular elongation varies according to the macular retinal layers. Liu et al. [34] reported that eyes with high myopia showed a thinner inner nuclear layer (INL) compared to those without high myopia. INL thinning may result in a secondary decrement of the DVP mVD, which provides blood flow to the INL [35]. Therefore, DVP mVD may be more vulnerable in HMG than in NMG to the blood flow impairment associated with glaucomatous damage, leading to higher correlations with cVFS. Fourth, retinal tissue thinning occurs with axial elongation and/or glaucomatous damage in HMG, and this may reduce the oxygen supply, leading to impaired blood circulation and further narrowing of the retinal VD [36,37]. As smaller vessels mainly constitute the VD in the deep retinal layer rather than the superficial retinal layer, the DVP mVD is more readily attenuated compared to the SVP mVD when axial elongation progresses as in our HMG eyes [36,38].

The linear regression analyses in our present investigation examined various factors associated with cVFS in each OAG subgroup, classified by the amount of axial elongation. The results of the multivariable analysis showed that older age, thinner mGCIPLT, lower SVP, and DVP mVD were significantly associated with cVFS loss in the HMG eyes, whereas older age, thinner mGCIPLT, and lower SVP mVD were associated with cVFS loss in the NMG eyes (Table 3). It is known that the sensitivity threshold values of the VF test decrease with increasing age. Haas et al. [39] reported that the differential light sensitivity decreases gradually throughout life at a rate of 0.58 dB per decade. Spry et al. [40] revealed that the mean VF sensitivity loss rate increased from 0.43 dB/decade to 1.02 dB/decade after 53.4 years. These previous findings are consistent with our current results, which indicated a significant association between older age and lower cVFS, regardless of the presence of high myopia.

A reduced SVP and DVP mVD were found in our present analysis to be independently associated with cVFS loss, after adjusting for age and mGCIPLT in HMG eyes, while only reduced SVP mVD showed this association in NMG eyes (Table 3). These findings support our hypothesis that mVD loss vs. cVFS reduction relationship may differ according to the location of retinal layers in HMG and NMG eyes, in which the DVP mVD may have a close link to cVFS in HMG eyes. In support of this possibility, Lin et al. [11] previously compared the reduction rates for the SVP mVD and DVP mVD in OAG eyes with or without high myopia. These authors revealed that the DVP mVD reduction rate of highly myopic eyes was significantly faster, whereas there was no significant difference in the SVP mVD reduction rate between the two groups.

There were several limitations of our present study of note. First, corrections of the ocular magnification effects on the mVD measurements via OCT-A were not conducted during our analyses. Sampson et al. [18] have reported however that mVD measurements are minimally affected by the ocular magnification related to axial elongation since the distribution of the microvascular network is relatively uniform in the parafoveal area. In our present study, we evaluated the mVD in the area that mainly included the parafoveal region. Second, despite the utilization of PAR algorithms to minimize the projection flow artifacts from the superficial retinal layer, the DVP mVD measurements obtained using the current OCT-A device may have been affected by the residual projection artifacts in both HMG and NMG groups. Since eyes with high myopia are associated with lower VD measurements in the layers of SVP and DVP, the impact of projection artifacts from the superficial retina may have been greater in NMG eyes than in HMG eyes. Of note, however, despite the denser SVP mVD in healthy eyes than in NMG eyes, there was no significant difference in the DVP mVD between these two groups, suggesting that projection flow might not have had a great impact on our results. Third, ocular hypotensive eye drops or systemic hypotensive medications may affect ocular blood flow [8,19]. Therefore, our findings should be interpreted with consideration of the possible compounding effects of topical and/or systemic hypotensive medication on the OCT-A measurements. Fourth, we recruited only Korean patients who visited our university hospital. As patients in a tertiary referral practice may have different characteristics than those observed in the general population, our results should be cautiously interpreted in terms of their overall clinical applicability, including in relation to other ethnic populations. Lastly, due to our cross-sectional study design, we could not provide information on the temporal relationship between SVP/DVP mVD and cVFS changes in each OAG group. This association needs to be investigated in future prospective longitudinal studies. Fifth, even though normal healthy participants served as a control group in the current study, it would have been more interesting to include a healthy myopic group for comparisons of various clinical data among different diagnostic groups. Sixth, the 10-2 VF test may be more appropriate for evaluation of the relationships of mVD and mGCIPLT with cVFS, as it provides more information about central VF damage and higher sensitivity for cVFS loss than 24-2 VF tests in patients with macular damage [41]. However, 24-2 VF tests are frequently used to test for central VF damage in the routine clinical setting. Further studies using 10-2 VF tests may provide more accurate information on the vasculature-function relationship.

In conclusion, global and regional DVP mVD–cVFS correlations are comparable to mGCIPLT–cVFS or SVP mVD–cVFS correlations in OAG eyes with high myopia but are weak in eyes without high myopia. In addition, a DVP mVD reduction, as well as SVP mVD/mGCIPLT reduction, is significantly associated with cVFS loss in OAG eyes with high myopia, suggesting that DVP mVD may have the potential to serve as a complementary tool for monitoring cVFS loss in OAG patients with high myopia.

## Figures and Tables

**Table 1:** Demographic and clinical characteristics of the study subjects.

	A. High Myopia (*n* = 64)	B. Non-High Myopia (*n* = 84)	C. Normal Eyes (*n* = 54)	*p*-Value	Post-Hoc (A vs. B, A vs. C, B vs. C)
Age, years	51.70 ± 11.78	54.48 ± 9.27	55.80 ± 9.94	0.085	-
Sex, male:female	41:23	34:50	25:29	**0.015**	**0.012**, 0.128, 0.777
Diabetic mellitus, *n* (%)	4 (6.2)	6 (7.1)	3 (5.5)	0.517	-
Hypertension, *n* (%)	11 (17.2)	22 (26.2)	10 (18.5)	0.560	-
Number of anti-glaucoma medications, *n*	0.9 ± 0.9	1.1 ± 1.0	0.0 ± 0.0	**<0.001**	0.484, **<0.001**, **<0.001**
Intraocular pressure, mmHg	13.86 ± 2.34	13.96 ± 2.28	14.94 ± 3.11	**0.039**	0.967, 0.054, 0.070
Spherical equivalent, Diopter	−7.28 ± 2.44	−0.66 ± 1.80	−0.86 ± 2.00	**<0.001**	**<0.001**, **<0.001**, 0.855
Axial length, mm	27.39 ± 1.58	23.75 ± 0.79	24.15 ± 0.87	**<0.001**	**<0.001**, **<0.001**, 0.169
Central corneal thickness, µm	535.24 ± 41.90	538.75 ± 34.60	537.49 ± 36.01	0.861	-
VF Mean deviation, dB	−5.97 ± 5.10	−5.59 ± 4.11	0.39 ± 0.95	**<0.001**	0.829, **<0.001**, **<0.001**
Central VF Mean sensitivity, 1/L	1194.32 ± 593.08	1103.82 ± 450.49	2009.39 ± 498.49	**<0.001**	0.537, **<0.001**, **<0.001**
Ganglion cell inner plexiform layer, µm					
Global	65.93 ± 8.85	71.60 ± 7.56	81.31 ± 5.27	**<0.001**	**<0.001**, **<0.001**, **<0.001**
ST	66.68 ± 10.13	71.74 ± 9.64	80.54 ± 4.95	**<0.001**	**<0.001**, **<0.001**, **<0.001**
SC	68.93 ± 10.99	75.80 ± 9.54	81.81 ± 6.56	**<0.001**	**<0.001**, **<0.001**, **0.001**
SN	70.65 ± 11.78	79.60 ± 9.43	83.74 ± 5.89	**<0.001**	**<0.001**, **<0.001**, **0.030**
IN	68.09 ± 10.79	73.28 ± 10.53	81.33 ± 6.98	**<0.001**	**0.007**, **<0.001**, **<0.001**
IC	60.58 ± 11.11	66.01 ± 11.24	78.70 ± 6.87	**<0.001**	**0.006**, **<0.001**, **<0.001**
IT	60.95 ± 11.72	63.81 ± 11.30	80.26 ± 7.29	**<0.001**	0.255, **<0.001**, **<0.001**
Superficial Vascular Plexus, %					
Global	38.26 ± 7.53	42.12 ± 5.59	50.78 ± 5.06	**<0.001**	**0.001**, **<0.001**, **<0.001**
ST	37.06 ± 7.30	42.21 ± 5.45	49.91 ± 5.15	**<0.001**	**<0.001**, **<0.001**, **<0.001**
SC	40.22 ± 7.59	43.90 ± 5.67	51.70 ± 5.35	**<0.001**	**0.001**, **<0.001**, **<0.001**
SN	39.27 ± 8.21	43.15 ± 5.74	51.78 ± 5.21	**<0.001**	**0.001**, **<0.001**, **<0.001**
IN	39.95 ± 8.41	43.68 ± 6.10	51.88 ± 5.10	**<0.001**	**0.003**, **<0.001**, **<0.001**
IC	38.27 ± 7.55	41.67 ± 7.26	50.95 ± 5.38	**<0.001**	**0.010**, **<0.001**, **<0.001**
IT	35.22 ± 7.56	38.75 ± 6.86	48.68 ± 5.67	**<0.001**	**0.006**, **<0.001**, **<0.001**
Deep Vascular Plexus, %					
Global	44.91 ± 9.34	54.54 ± 9.70	56.05 ± 6.55	**<0.001**	**<0.001**, **<0.001**, 0.575
ST	46.27 ± 9.78	54.99 ± 8.97	56.88 ± 6.69	**<0.001**	**<0.001**, **<0.001**, 0.429
SC	44.91 ± 9.34	53.62 ± 9.33	55.15 ± 6.54	**<0.001**	**<0.001**, **<0.001**, 0.571
SN	45.07 ± 9.52	53.90 ± 8.86	55.30 ± 5.93	**<0.001**	**<0.001**, **<0.001**, 0.607
IN	44.18 ± 9.98	53.98 ± 9.28	55.40 ± 6.51	**<0.001**	**<0.001**, **<0.001**, 0.631
IC	43.90 ± 10.08	53.11 ± 9.66	54.95 ± 7.34	**<0.001**	**<0.001**, **<0.001**, 0.489
IT	44.81 ± 10.49	54.06 ± 9.63	56.46 ± 7.10	**<0.001**	**<0.001**, **<0.001**, 0.306

Statistically significant differences are indicated in bold. dB decibel, IN inferior nasal, IC inferior center, IT inferior temporal, L Lambert, ST superior temporal, SC superior center, SN superior nasal, VF visual field.

**Table 2:** Comparison of the correlation coefficients between the macular ganglion cell-inner plexiform layer thickness and macular vessel density parameters with the central visual field sensitivity.

All Subjects (*n* = 148)	mGCIPLT	SVP mVD	DVP mVD	Pairwise Comparison *p*-Values		
				*p* ^1^	*p* ^2^	*p* ^3^
Average	0.483 (**<0.001**)	0.509 (**<0.001**)	0.307 (**<0.001**)	0.669	0.039	**<** **0.001**
ST (IN cVFS)	0.502 (**<0.001**)	0.350 (**<0.001**)	0.253 (**0.002**)	0.035	**0.005**	0.050
SC+SN (IT cVS)	0.462 (**<0.001**)	0.476 (**<0.001**)	0.241 (**0.003**)	0.840	**0.013**	**0.001**
IC+IN (ST cVFS)	0.466 (**<0.001**)	0.441 (**<0.001**)	0.219 (**0.008**)	0.708	**0.005**	**<** **0.001**
IT (SN cVFS)	0.622 (**<0.001**)	0.563 (**<0.001**)	0.301 (**<0.001**)	0.297	**<** **0.001**	**<** **0.001**
High myopia (*n* = 64)						
Average	0.537 (**<0.001**)	0.572 (**<0.001**)	0.508 (**<0.001**)	0.705	0.795	0.284
ST (IN cVFS)	0.507 (**<0.001**)	0.535 (**<0.001**)	0.467 (**<0.001**)	0.781	0.734	0.220
SC+SN (IT cVS)	0.568 (**<0.001**)	0.501(**<0.001**)	0.423 (**<0.001**)	0.500	0.207	0.256
IC+IN (ST cVFS)	0.411 (**<0.001**)	0.512 (**<0.001**)	0.403 (**<0.001**)	0.361	0.950	0.121
IT (SN cVFS)	0.535 (**<0.001**)	0.518 (**<0.001**)	0.454 (**<0.001**)	0.874	0.520	0.309
Non-high myopia (*n* = 84)						
Average	0.552 (**<0.001**)	0.535 (**<0.001**)	0.270 (**0.013**)	0.835	0.030	**0.001**
ST (IN cVFS)	0.508 (**<0.001**)	0.176 (0.109)	0.076 (0.493)	**0.002**	**0.002**	0.272
SC+SN (IT cVS)	0.499 (**<0.001**)	0.498 (**<0.001**)	0.164 (0.137)	0.992	**0.015**	**<** **0.001**
IC+IN (ST cVFS)	0.631 (**<0.001**)	0.490 (**<0.001**)	0.259 (**0.018**)	0.070	**0.002**	**0.010**
IT (SN cVFS)	0.728 (**<0.001**)	0.702 (**<0.001**)	0.327 (**0.002**)	0.633	**<0.001**	**<0.001**

*p*^1^: mGCIPL–cVFS vs. SVP–cVFS; *p*^2^: mGCIPL–cVFS vs. DVP–cVFS; *p*^3^: SVP–cVFS vs. DVP–cVFS. Statistically significant differences are indicated in bold (*p* < 0.017). cVFS central visual field sensitivity, DVP deep vascular plexus, IN inferior nasal, IC inferior center, IT inferior temporal, mGCIPLT macular ganglion cell-inner plexiform layer thickness, mVD macular vessel density, SVP superior vascular plexus ST superior temporal, SC superior center, SN superior nasal, VF visual field.

**Table 3:** Univariable and multivariable linear regression analyses to identify clinical variables associated with central visual field sensitivity.

Total (*n* = 148)	Univariable Analysis			Multivariable Analysis Including SVP mVD			Multivariable Analysis Including DVP mVD		
	B	95% CI	*p*-Value	B	95% CI	*p*-Value	B	95% CI	*p*-Value
Age, years	−22.141	−29.131 to −15.150	**<0.001**	−20.466	−26.390 to 14.542	**<0.001**	−21.910	−27.967 to 15.854	**<0.001**
IOP, mmHg	8.736	−29.018 to 46.490	0.648						
CCT, µm	1.026	−1.285 to 3.337	0.381						
SE, diopters	−14.408	−36.717 to 7.901	0.204						
AL mm	12.553	−28.184 to 53.290	0.610						
mGCIPLT, µm	29.487	20.413 to 38.561	**<0.001**	18.464	8.812 to 28.117	**<0.001**	28.848	21.090 to 36.606	**<0.001**
SVP mVD, %	38.892	28.117 to 49.668	**<0.001**	20.659	8.526 to 32.793	**<0.001**			
DVP mVD, %	15.654	7.947 to 23.361	**<0.001**				Dropped		
High myopia (*n* = 64)									
Age, years	−26.527	−37.385 to −15.670	**<0.001**	−20.775	−30.567 to 10.982	**<0.001**	−21.250	−30.940 to 11.561	<0.001
IOP, mmHg	25.359	−41.589 to 92.307	0.452						
CCT, µm	0.521	−3.285 to 4.326	0.785						
SE, diopters	−0.329	−49.913 to 49.256	0.989						
AL mm	−24.013	−122.437 to 74.411	0.627						
mGCIPLT, µm	37.261	21.452 to 53.070	**<0.001**	20.458	4.743 to 36.174	**0.012**	24.685	10.640 to 38.730	0.001
SVP mVD, %	45.218	28.769 to 61.667	**<0.001**	22.932	4.677 to 41.188	**0.015**			
DVP mVD, %	30.656	17.454 to 43.858	**<0.001**				15.537	3.336 to 27.738	0.014
Non-high myopia (*n* = 84)									
Age, years	−17.405	−27.245 to −7.565	**0.001**	−14.934	−22.852 to 7.015	**<0.001**	−15.590	−24.080 to 7.820	**<0.001**
IOP, mmHg	−2.874	−46.636 to 40.889	0.896						
CCT, µm	1.682	−1.200 to 4.565	0.249						
SE, diopters	−35.119	−90.392 to 20.154	0.210						
AL. mm	−14.4684	−146.295 to 117.360	0.827						
mGCIPLT, µm	32.730	21.647 to 43.813	**<0.001**	21.672	9.289 to 34.055	**0.001**	31.597	21.378 to 41.816	**<0.001**
SVP mVD, %	42.899	27.938 to 57.860	**<0.001**	23.007	6.012 to 40.002	**0.009**			
DVP mVD, %	12.523	2.701 to 22.344	**0.013**	Dropped					

The multivariable model was applied with stepwise elimination. Statistically significant differences are indicated in bold. AL axial length, B β-coefficient, CCT central corneal thickness, CI confidence interval cVFS central visual field sensitivity, DVP deep vascular plexus, IOP intraocular pressure, mGCIPLT macular ganglion cell-inner plexiform layer thickness, mVD macular vessel density, SE spherical equivalent, SVP superior vascular plexus.

## Data Availability

Data collected for this study, including individual patient data, will not be made available.
